# Nursing Guidelines on Cardiac Surgery and Parents’ Anxiety: Randomized Clinical Trial

**DOI:** 10.21470/1678-9741-2019-0345

**Published:** 2020

**Authors:** Ilsa Beatriz Machado Xavier, Virginia Borne Biscarra, Ângela Bein Piccoli, Clarissa Garcia Rodrigues, Vania Naomi Hirakata, Maria Antonieta Pereira de Moraes, Lucia Campos Pellanda

**Affiliations:** 1 Instituto de Cardiologia/Fundação Universitária de Cardiologia - IC/FUC, Porto Alegre, RS, Brazil.; 2 Universidade Federal de Ciências da Saúde de Porto Alegre, Porto Alegre, RS, Brazil.; 3 Hospital de Clínicas de Porto Alegre, Porto Alegre, RS, Brazil.

**Keywords:** Preoperative Period, Control Groups, Anxiety Disorders, Parents, Personality Inventory, Cardiac Surgical Procedures, Analysis of Variance

## Abstract

**Objective:**

The preparation of parents of children who should undergo cardiac surgery requires special treatment such as the explanations about the event. This study aims to compare the effects of standardized nursing guidelines with routine institutional orientation on the anxiety of parents of children undergoing cardiac surgery.

**Methods:**

Randomized clinical trial. The sample consisted of parents of children who underwent cardiac surgery from December 2010 to April 2011. Twenty-two parents were randomized to the intervention group (IG) and received the standard nursing guidelines and 22 participated in the control group (CG) and received the routine guidelines from the institution. Anxiety was assessed by the State-Trait Anxiety Inventory (STAI) applied in the preoperative period, between 12 and 20 hours before surgery and before receiving standard or institutional guidelines and 48 hours after surgery. The analysis of variance (ANOVA) for repeated measures was performed to evaluate the differences between the variations in STAI scores between the groups during the studied period. The level of significance was 0.05.

**Results:**

There were no significant differences in baseline anxiety scores between groups with regard to trait anxiety as well as state anxiety: STAI-trait (CG 42.6±4.9 *vs*. IG 41.4±6.0, *P*=0.48); STAI-state (CG 42.3±5.7 *vs*. IG 45.6±8.3, *P*=0.18). Likewise, the variation in score after 48 hours was similar between groups (STAI-trait *P*=0.77; STAI-state *P*=0.61).

**Conclusion:**

There were no significant differences in the parents’ anxiety levels when comparing the two types of guidelines: the standard nursing and the institutional orientation.

**Table:** 

Abbreviations, acronyms & symbols	
ANOVA	= Analysis of variance
CG	= Control group
CHD	= Congenital heart disease
ICU	= Intensive care unit
IG	= Intervention group
SPSS	= Statistical Package for the Social Sciences
STAI	= State-Trait Anxiety Inventory

## INTRODUCTION

Cardiac malformations are considered one of the most frequent forms of congenital anomaly, comprising about 25% of congenital malformations^[1]^ or about 10:1000 live newborns are affected by some type of congenital heart anomaly, with 1/3 of diagnoses requiring surgical intervention^[2]^. In the Brazilian population, a study identified the same proportion, from 8 to 10:1000 live births^[1]^, close to the prevalence presented by Asia^[2]^ and it is estimated that about 29,000 new cases of congenital heart disease (CHD) occur each year in Brazil, of which 20% have spontaneous resolution^[1]^.

Among the treatments available, cardiac surgery is one of the strategies for the most complex cases^[1]^ and its recognition is important given the rapid clinical deterioration and high mortality, as about 20 to 30% of children with CHD die in the first month of life due to heart failure or hypoxia attacks^[3]^. In Brazil, an average of 23,077 surgical procedures are performed per year, 50% of them in the first year of life^[1]^.

The family undergoes major changes and is subject to a significant emotional impact during the hospitalization of one of its members, especially if it is a child. In this context, the expression of anguish, fear and anxiety, suffering and uncertainties is common in parents of children undergoing cardiac surgery, due to the severity of the situation and the limited knowledge regarding trans and postoperative periods. Studies point anxiety as a negative emotion, accompanied by distinct psychological and somatic attributes, with an adaptive purpose of fighting for survival. It is also described as “apprehensive anticipation of future danger or misfortune accompanied by a feeling of dysphoria or somatic symptoms of tension”^[4]^ and the focus of the anticipated danger can be both internal and external to the person. Freud (1925) understood anxiety as a normal reaction to an external danger that causes a state of increased motor tension or paralysis, causing more anxiety. Anxiety produces confusion and perceptual distortions in terms of time, space, people and meanings of events^[5]^.

Surgery has a major impact on the patient’s physical, social and emotional well-being, with increased levels of anxiety and stress due to the temporary distance from the family support network and also to the potential benefits of its use^[6]^. Parents and family members are closely involved in the diagnosis, treatment and rehabilitation process, and therefore the psychological preparation of patients and the closest family network is important. According to the complexity of the disease and the surgical procedure, such as cardiac surgery in children, which in some cases, two or more interventions are associated in the same surgical stage, the need for the healthcare professional to assist the patient or the parents of patients dealing with anxiety implies offering very specific orientations on the surgical process and hospitalization^[7]^. The specificities of cardiac pathologies and the psychological aspects involved trigger popular fantasies and beliefs about the disease that may interfere with the way family members face surgical intervention. The anxieties and anguish commonly associated with heart disease are those related to death^[5]^ and it is necessary to develop different orientation strategies to minimize this feeling.

The nurse is considered a specialized professional and has the task of including the parents in the child’s healthcare, especially when undergoing a hospital procedure. Nursing care is very comprehensive, from hospitalization to discharge, including guiding, conveying safety and allowing patients and families to express their feelings, anxieties and anguish.

Systematized and standardized nursing strategies for specific explanations to the parents of children with CHD on the verge of surgical procedures may help reduce their anxiety. There are studies that address nursing interventions directly in patients’ anxiety^[7]^, but no clinical trials have been found so far to verify this hypothesis in parents of children undergoing cardiac surgery. This study aims to evaluate the impact of standardized nursing guidelines on the anxiety of parents of children undergoing cardiac surgery compared to institutional orientation.

## METHODS

This study is a randomized clinical trial designed according to guidelines and norms that regulate research involving human beings and was approved by the Research Ethics Committee of the Rio Grande do Sul Institute of Cardiology/University Foundation, according to the Ministry of Health resolution 196/96, in force during the study period. The study population consisted of parents of children who underwent cardiac surgery from December 2010 to April 2011. Parents over the age of 18 and who agreed to participate in the study were included. Parents of children who underwent implantation of pacemaker and implantable cardioverter defibrillator, or parents unable to understand and/or answer questions and surgical death, were excluded.

To calculate the sample size, a 5% alpha error, a 20% beta error and a standard deviation of the STAI-S score of 9.9 were considered^[8]^. To obtain a reduction in state-anxiety levels whose standardized effect size was ≥ 0.9 (considered strong)^[9,10]^, a sample of at least 40 participants was estimated, 20 for each group.

### Randomization

An active search of children with scheduled elective surgery or admitted in inpatient units of the institution was performed. Once the surgical procedure was confirmed, parents were invited to participate in the study with adequate clarification. Those who accepted signed the free and informed consent form. Patients were randomized into Control Group (CG) and Intervention Group (IG). The randomization list was generated at www.randomization.com, using a block of 44 numbers which were randomized into two groups of 22 numbers. The list was made by a professional outside the study. The researchers had no contact with the list at any time in the study. The study was registered under National Clinical Trial (NCT) number 01492452.

### Measurements

A sociodemographic questionnaire was applied, which included questions related to data of parents and children. Among the questions being considered were the age of the children, previous surgeries, history of another child with heart disease and if the parents considered use of medication for anxiety in a systematic way by medical indication.

For the evaluation of parents’ anxiety, the State-Trait Anxiety Inventory (STAI) was defined, and the application was guided by psychologists. This inventory was developed in 1970 by Spielberger, Gorsuch and Lushene^[11]^ and translated and adapted for the Brazilian population by Biaggio in 1979^[12]^ after an experimental scale development of the study in Portuguese carried out in 1977^[8]^. It is a self-report questionnaire composed of two distinct scales to measure two dimensions of anxiety: the anxious state (STAI-S) and the anxious trait (STAI-T). Each scale consists of twenty statements for which the volunteers indicate the intensity at that time (STAI-S) or the frequency with which they occur (STAI-T) using a four-point Likert scale (1 to 4). The total score for each scale ranges from 20 to 80, with higher values indicating higher levels of anxiety. According to the criteria established in the method, the cutoff points for STAI-S were: low anxiety: < 35; moderate anxiety: 36 to 46; high anxiety: > 47; and for STAI-T: low anxiety: < 32; moderate anxiety: 33 to 41; high anxiety: > 42^[13]^.

The scales were applied in two moments: 1) the institution’s surgery schedule is informed at 5 pm on the day before surgery, when it is confirmed which patients will undergo surgery. Thus, the researchers had access to this agenda at the time and applied the instrument for the first time when parents also had the day and time of surgery confirmed. Following this application, a telephone contact was made with the professional who had the randomization list, to find out the group to which the patient would belong, CG or IG, so that the standardized nursing guidelines to the IG were made; 2) the second application was performed 48 hours after the children’s cardiac surgery. The anxiety scale data were collected and analyzed by the statistician, not involved in the application of the instrument or in the guidelines.

### Intervention and Control

**Fig. 1:**
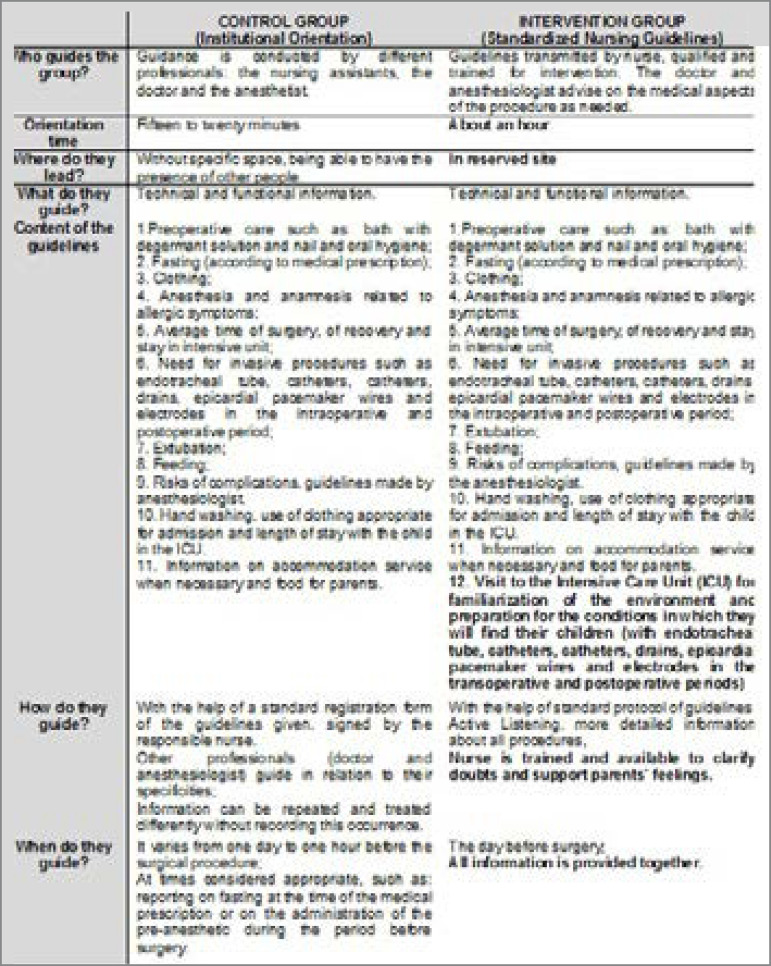
Reference framework for intervention comparison.

Figure 1 shows the comparative image of the interventions. The basic difference between the two interventions is: 1) standard protocol; 2) inclusion of a visit to the intensive care unit (ICU) environment; 3) orientation time; 4) location and specific moment; 5) active listening.

The standardized guidelines are understood as a specific step that integrates the period between preoperative, intraoperative and postoperative periods. In a scheme, we can express it as follows:

1. Institution guidelines
  Preoperative → Intraoperative → Postoperative (basic guidelines throughout the process, at specific moments, by several nursing professionals)
2. Standardized guidelines
  Preoperative → Basic guidelines and ICU visit → Introperative → Postoperative (guidelines made by the nurse in charge)

### Statistical Analysis

The statistical program used was SPSS, version 23. Variables were described as mean and standard deviation for continuous variables and absolute and relative frequencies for categorical variables. The distribution of variables regarding normality was evaluated by the Shapiro-Wilk test. Comparisons between groups were performed using Student’s t-test for independent samples for continuous variables and Pearson’s chi-square test for categorical variables. Analysis of variance (ANOVA) for repeated measures was performed to evaluate the differences between variations in STAI scores between the groups, over the studied period. The level of significance was 0.05.

## RESULTS

**Fig. 2:**
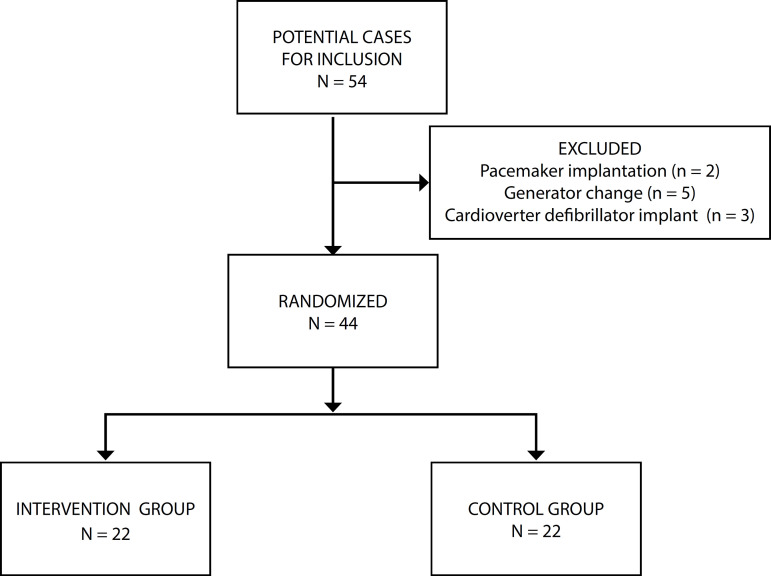
Flowchart of study participants.

Between December 2010 and April 2011, 54 parents of potentially eligible children were assessed for inclusion in the study. Among them, 10 were excluded due to some previously established exclusion criteria. A total of 44 parents were randomized, 22 of whom received standardized nursing guidelines (IG) and 22 received routine institution guidelines (CG) (Figure 2).

The characteristics of the sample were similar in both groups, as shown in Table 1. The mean age of parents was 34.6±10.6 years in the IG and 39.5±16.3 in the CG; with prevalence of the mother in both groups, 90.9% in the IG and 95.5% in the CG; most of the parents, both IG and CG, have an income between 1 and 3 minimum wages and attended high school; 9.1% of the parents of the IG and 27.3% of the parents of the CG reported use of medication for anxiety systematically. In the IG, 13.6% of the parents and 4.5% of the CG have another child diagnosed with heart disease. Among the IG parents, 31.8% have experienced at least one previous heart surgery in their child. As for the age of children who were in surgery, it ranged from 2 months of age to 15 years. For the purposes of analysis, the sample was divided into two age groups, considering the baby phase from 0 to 2 years and the second group from 2.1 to 15 years.

**Table 1:** Characteristics of the population of the studied groups*.

Characteristics		Intervention Group (n=22) n (%)	Control Group (n=22) n (%)	*P*-value
Parents's age**		34.6 ± 10.6	39.5 ± 16.3	0.24
Children's age	0-2 years old	9 (59.1)	13 (40.9)	0.22*
	2.1-15 years old	13 (40.9)	9 (59.1)	
	Female gender	20 (90.9)	21 (95.5)	1*
Family income (MW)***	≤1	5 (22.7)	6 (27.3)	0.75*
	01-Mar	12 (50.0)	13 (59.1)	
	03-Jun	4 (18.2)	2 (9.1)	
	06-Oct	1 (4.5)	1 (4.5)	
	>10	1 (4.5)	-	
Parent's schooling	Elementary school	8 (36.4)	8 (36.4)	0.63*
	High school	11 (50.0)	10 (45.5)	
	Higher education	2 (9.1)	4 (18.2)	
	Postgraduation	1 (4.5)	-	
	Previous diagnosis of anxiety	2 (9.1)	6 (27.3)	0.12*
	Another child with heart disease	3 (13.6)	1 (4.5)	0.61*
	Children with previous surgery	6 (7.27)	7 (31.8)	0.74*

* Comparison with the Pearson chi-square test.

** Variable described in mean and standard deviation, compared by Student's t-test.

*** MW = current minimum wage corresponding to BRL 546.57 in the period.

Table 2 shows the mean of the state-trait anguish and anxiety scores before and after the nursing orientations, considering the mean of the two groups (IG and CG). The results related to the dimension of anxiety trait (STAI-T) and anxiety state (STAI-S) revealed that in both groups, the average anxiety classified as ‘moderate’ before and after the intervention, according to established cutoff points^[16]^, the difference is not significant. The variation in scores in the study period after 48 hours was similar between the groups (STAI-T *P*=0.77; STAI-S *P*=0.61).

**Table 2:** Averages of anxiety trait and anxiety state levels before and after nursing orientation.

Type of anxiety	Groups	N	Preorientation m (sd)	Postorientation (sd)	Average variation (IG 95%)	*P*-value*
Trait	CG	22	42.6 (4.9)	40.8 (6.3)	-1.82 (-4.6;0.9)	0.482
(STAI-T)	IG	22	41.4 (6.0)	40.9 (4.9)	-0.46 (-3.2;2.3)	
	Total	44	42.0 (5.5)	40.8 (5.5)		
State	CG	22	42.3 (5.7)	42.4 (5.5)	0.18 (-2.2;2.6)	0.186
(STAI-S)	IG	22	45.6 (8.3)	43.5 (6.1)	-2.09 (-4.5;0.3)	
	Total	44	43.9(7.3)	43.0 (5.8)		

Note: the P-value corresponds to the interaction term (groups x moment) of ANOVA for repeated measures.

To determine if previous cardiac surgery in children could influence the anxiety levels in both groups (IG and CG), a comparative analysis was made between parents who had previous experience of cardiac surgery in the child and those who did not have this experience. The means were similar both before and after the guidelines (Table 3).

**Table 3:** Comparison of the means of anxiety-state in parents with and without previous cardiac surgery experience in the child.

	Parents without previous experience of surgery		Parents with previous experience of surgery	
	CG (n=15)	IG (n=16)	CG (n=7)	IG (n=6)
Anxiety state	m (sd)	m (sd)	m (sd)	m (sd)
Before orientations	43.6 (5.1)	44.1 (8.7)	39.4 (6.3)	49.7 (6.3)
After orientations	43.7 (5.3)	43.1 (6.3)	39.7 (5.3)	44.5 (6.1)

In the same way, an analysis was carried out, considering whether the children’s age group could be a factor that justified anxiety state levels, before and after surgery. Two ranges are defined: babies from 0 to 2 years old, and children from 2 to 15 years old. There was no significant difference (*P*=0.29) between anxiety state, before intervention, between parents of children aged 0 to 2 years: 43.5 (±5.1) *versus* parents of children aged 2.1 to 15 years: 44.8 (±3.2). Likewise, there were no significant differences (*P*=0.81) between the anxiety state, after intervention, of parents of children aged 0 to 2 years 44.5 (±5.7) *versus* parents of children aged 2.1 to 15 years: 41.4 (±0.5.6).

## DISCUSSION

An important aspect in the psychological preparation of the family is the effective preoperative orientation, which allows to reduce the parental anxiety and, consequently, the psychological responses to stress before and after surgery by the children^[14]^. Researchers deepened their understanding of the attribute anxiety in two factors: anxiety trait and anxiety state^[15,16]^. The anxiety state reflects a reaction related to a situation of adversity that presents itself at a given moment, which depends on environmental stimuli and, therefore, varies in intensity and refers to acute situations. It is a transient emotional condition consisting of feelings of tension, apprehension, nervousness, worry, and increased activity of the autonomic sympathetic nervous system. The anxiety trait refers to a more stable aspect, related to the individual’s propensity to deal with greater or lesser anxiety throughout life. It is related to relatively stable individual differences in the quality of behavioral responses. The anxiety trait characterizes individuals hypersensitive to stimuli and more psychologically reactive.

The results of this study showed that the anxiety state of the parents of children who underwent cardiac surgery in a hospital specialized in cardiology did not change significantly between the preoperative and postoperative periods, after the standardized nursing orientations. That is, there were no significant differences in reducing anxiety in any of the groups that demonstrated that standardized nursing guidelines could reduce parental anxiety more than routine institutional orientation.

These findings are related to the later studies of Spielberger (1982), who recognized the difficulty of interpretation regarding the nature of anxiety, especially anxiety trait, establishing two other factors related to the presence or absence of anxiety. The presence of anxiety would be associated with negative feelings related to worries, tensions and insecurity, whereas items associated with the factor “absent anxiety” would describe the presence of positive feelings of well-being, satisfaction and happiness^[20]^. Other studies, however, have shown that items related to mood states, such as crying, depression and happiness, were a factor regardless of whether they were associated with items that expressed the presence or absence of anxiety, being more adjusted to aspects of depression^[17]^. In this sense, although anxiety and depression are distinct constructs, they express themselves in a similar way and, for this reason, the evaluation of anxiety has been considered a challenge.

Previous studies evaluating preoperative psychological preparation interventions to reduce anxiety have shown controversial results, some demonstrating the effectiveness of the intervention and others not identifying differences between groups. For example, nursing interventions, such as standardized guidelines, have had satisfactory results in reducing anxiety in patients undergoing abdominal surgery^[18]^. Several variables are involved in coping with anxiety (demographic, biological and cognitive partners). In the context of CHD, however, other factors triggering preoperative anxiety are considered: early perception of pain and discomfort; passive waiting for the procedure to begin; separation from family or child; feelings of abandonment; loss of autonomy; fear of imminent death, sequelae, anesthesia and surgical procedure as a whole^[16]^.

Most studies aim to analyze methods to reduce the anxiety of the adult patients themselves. In the absence of studies with parents of cardiac children to compare the results, we used as a comparison parameter studies that used systematic and standardized interventions to reduce anxiety, even though the focus of the studies were adult patients, understanding that parents might be experiencing similar situations to these patients. For example, a study at a cardiology hospital that aimed to reduce the feelings of fear and anxiety among 60 adult patients undergoing coronary artery bypass grafting showed that patients, when oriented in groups, presented reduction in levels of anxiety and fear when compared to patients oriented individually according to the institutional routine^[19]^. This study revealed important differences, as the preparation of the patients was in groups, allowing the sharing of anxieties.

Another study, which compared two types of orientations to adult cardiac patients in the preoperative period, in which one group received medical information through brochures and another group received a combination of extensive oral information, accompanied by the surgeon’s attention in the preoperative period, detected high levels of stress during transport to the operating room in both groups; which meant that extensive oral information in the preoperative period plus the physician’s attention had no significant influence on the ‘somatic’ symptoms of preoperative stress. This study showed that the results did not identify differences between the approaches, but there was reduction in anxiety in both groups after surgery^[20]^. The same happened with our investigation; the fact that parents receive more attention, with more detailed guidance and exclusive care from nurses, did not significantly reduce anxiety when compared to those who received the guidelines at different times, with different professionals. It can be understood that the concerns and fears related to this type of surgery, including the real possibility of the child’s death, are so intense that they resist the information received or prior knowledge as well as confidence in the medical staff, while the patient is not free of risk.

Similar results were analyzed in another study (Shuldham, 2002) that used “preoperative education” in the admission of patients (188 in the experimental group *vs*. 168 in the control group) who assessed pain, anxiety, depression, and several days after myocardial revascularization surgery (3 days, 6 weeks, 3 and 6 months) and did not identify differences between the groups regarding the outcomes^[21]^. This study, with objectives related to the appearance of symptoms and feelings beyond the postoperative period, also found no differences in groups that received a more careful preparation.

The use of relaxation techniques, the use of music, changes in the physical structure of the hospital environment and the development of activities by multidisciplinary teams were pointed out as complementary to reducing the patient’s preoperative anxiety, which would involve a more institutional service policy than only the nursing service.

Nursing has sought to integrate knowledge and skills, with the aim of improving the quality of care for the frailty of the family as a whole. Most studies focus on adult patients and not on parents or family members of children in the process of performing surgical procedures. The low variation in anxiety-state scores (STAI-S) can be attributed to several factors. One of them would be the high quality of the institution’s orientations, since it is a specialized hospital and a reference in cardiology, in which there is concern of all the interdisciplinary team in favor of patient recovery, in addition to the functional staff of nurses with specialization in cardiology, which may have contributed to similar results between groups.

Among the limitations of this study, we identified the difficulty of establishing the moment to measure the state of anxiety variation in the context of a surgical procedure, considered very complex. The first application of the anxiety verification instrument was defined after confirmation of the surgery to be performed the next day, followed by the intervention when the patient was identified as part of the intervention group, and the second application of the STAI was 48 hours after surgery (when the patient, in principle, has already left the most critical postoperative phase). It was assumed that standardized nursing information would be effective in reducing anxiety, but the non-significant difference in anxiety variation showed that, even with favorable conditions and positive postoperative outcomes, anxiety remained at levels similar to those of anxiety before surgery and guidelines. In this sense, considering the parents’ suffering in relation to their children’s heart disease, having to face surgery as the last and only resource for life, one might think that anxiety-state remains at similar levels in the moments when surgery is eminent fact, during surgery and after surgery. In this way, we can assume that it will be smoothed only after the conclusion of the whole process, that is, when the patient is discharged or beyond, as shown by the conclusions of Shuldham’s study (2002)^[21]^ above.

The intervention may have been beneficial so that aspects considered unfamiliar and “imagined” and sometimes with extreme negative connotations about surgery can be clarified, making parents face the situation with more awareness of the real. However, the fact that the child is still at risk or in need of care, anxiety, which is a fear of something unknown, remains at a level similar to that seen in the preoperative period, since other new possible issues related to the process emerge of recovery from surgery, which demonstrates that anxiety is a biological, dynamic, and ever-present feature in response to events that are about to occur.

A review study on the psychological preparation of patients undergoing surgery showed that most articles comprised the interventions that informed on the surgical procedure and the recovery process, taking into account patients’ general physical and psychosocial symptoms^22^. The information had the objective to qualify the patient with technical data and reducing anxiety symptoms, which occur more often when the subject is exposed to unknown situations and classified as potentially aversive. In this study^[22]^, they also found that health professionals need to be qualified to provide information that represents adequate psychological support to patients. In an attempt to reassure the patient, they may eventually provide information that increases the anxiety and fear of the person who will undergo surgery or their families. This issue may have occurred in relation to the ICU visit, where the scenario presents itself in an environment with many equipment and with patients in serious condition. Although the objective was to prepare the parents for the meeting with the child after the surgery, showing the real care environment, this environment for some may have an impact and the anxiety may have remained.

## CONCLUSION

There were no significant differences in parents’ anxiety levels when compared to the two modes of orientation: standardized nursing and institutional orientation in a cardiology hospital. The health professional should always seek new strategies and approaches to minimize parents’ anxiety and fragility. Providing more detailed guidance and expanding parents’ knowledge about the children’s conditions in the postoperative period, with a visit to the ICU, in an attempt to minimize anxiety of fantasies, was not enough. However, ongoing research is needed on what procedures health institutions - hospitals - should adopt so that parents, in situations of their children’s surgeries, can be more easily deal with adversity; group and video guidelines, for example, in preoperative and postoperative periods should be stimulated.

### Sources of Funding

The present study had no external sources of financing.

### Academic Link

This article is part of the conclusion work of the Multidisciplinary Residency Program in Cardiology Nursing.

**Table:** 

Authors' roles & responsibilities	
IBMX	Substantial contributions to the conception or design of the work; or the acquisition, analysis, or interpretation of data for the work; drafting the work or revising it critically for important intellectual content; final approval of the version to be published
VBB	Substantial contributions to the conception or design of the work; or the acquisition, analysis, or interpretation of data for the work; drafting the work or revising it critically for important intellectual content; final approval of the version to be published
ABP	Substantial contributions to the conception or design of the work; or the acquisition, analysis, or interpretation of data for the work; drafting the work or revising it critically for important intellectual content; final approval of the version to be published
CGR	Substantial contributions to the conception or design of the work; or the acquisition, analysis, or interpretation of data for the work; drafting the work or revising it critically for important intellectual content; final approval of the version to be published
VNH	Substantial contributions to the conception or design of the work; or the acquisition, analysis, or interpretation of data for the work; drafting the work or revising it critically for important intellectual content; final approval of the version to be published
MAPM	Substantial contributions to the conception or design of the work; or the acquisition, analysis, or interpretation of data for the work; drafting the work or revising it critically for important intellectual content; final approval of the version to be published
LCP	Substantial contributions to the conception or design of the work; or the acquisition, analysis, or interpretation of data for the work; drafting the work or revising it critically for important intellectual content; final approval of the version to be published
